# Serum Levels of Lipids and Selected Aminothiols in Epileptic Children—A Pilot Case-Control Study

**DOI:** 10.3390/brainsci12010120

**Published:** 2022-01-17

**Authors:** Beata Sarecka-Hujar, Izabela Szołtysek-Bołdys, Ilona Kopyta

**Affiliations:** 1 Department of Basic Biomedical Science, Faculty of Pharmaceutical Sciences in Sosnowiec, Medical University of Silesia in Katowice, 41-200 Sosnowiec, Poland; 2 Department of General and Inorganic Chemistry, Faculty of Pharmaceutical Sciences in Sosnowiec, Medical University of Silesia in Katowice, 41-200 Sosnowiec, Poland; iboldys@sum.edu.pl; 3 Department of Pediatric Neurology, Faculty of Medical Sciences in Katowice, Medical University of Silesia in Katowice, 40-752 Katowice, Poland; ilonakopyta@autograf.pl

**Keywords:** epilepsy, children, homocysteine, cysteine, glutathione, lipid levels

## Abstract

Background: Standard treatment of epileptic seizures involves the use of antiepileptic drugs (AEDs). Both AEDs themselves and treatment duration may influence the levels of biochemical parameters, e.g., lipids or homocysteine (HCys), that may increase the risk of cardiovascular diseases. The aim of the present study was to compare the levels of lipid parameters, as well as the concentrations of selected aminothiols (i.e., HCys, cysteine, and glutathione) between epileptic children treated with multiple AEDs and children without epilepsy. Methods: In the study, 21 children with epilepsy treated with two or more AEDs for at least 6 months (8 girls and 13 boys, mean age 7.03 ± 4.51) and 23 children without epilepsy (7 girls and 16 boys, mean age 7.54 ± 3.90) were prospectively analyzed. Lipid parameters, i.e., total cholesterol (TC), triglycerides (TG), low density lipoprotein (LDL) and high density lipoprotein (HDL), and levels of selected aminothiols were determined in the blood serum. Results: No differences in the mean levels of lipid parameters and in the mean values of lipid ratios (TC/HDL, TG/HDL, LDL/HDL) were observed between the total groups as well as in the sex subgroups. HCys and cysteine levels did not differ between the patients and controls. We observed significantly lower levels of glutathione in children with epilepsy than in children without epilepsy (1.49 ± 0.35 µmol/L vs. 2.39 ± 1.17 µmol/L, respectively) (*p* < 0.001). Glutathione level was also lower in boys with epilepsy than in boys without epilepsy (*p* = 0.007). Similarly, epileptic girls had statistically decreased levels of glutathione when compared to girls without epilepsy (*p* = 0.006). Conclusions: A lower level of glutathione is observed in pediatric patients with epilepsy treated with two or more AEDs for at least 6 months. This indicates the oxidative stress of the patients treated with AEDs, which in turn may affect their well-being, and in the case of chronic occurrence resulting from long-term treatment, also on the function of the liver and the condition of the cardiovascular system.

## 1. Introduction

The dynamic process of epileptogenesis involves one or several damaging factors including metabolic, environmental and genetic factors or trauma [1]. Standard treatment of epileptic seizures involves the use of antiepileptic drugs (AEDs). Monotherapy is the most recommended treatment for epileptic children. In order to achieve effective monotherapy for newly diagnosed epilepsy, it is necessary to: select the AED for the specific type of seizure; select an AED with a tolerable side effect and toxicity profile; and slowly titrate the selected AED to the desired dose, analyzing the patient’s response to treatment. In some refractory patients, presenting neurotoxic adverse effects with high-dose monotherapy, polytherapy with lower or moderate dosages of two AEDs may be sometimes preferred [2]. It is observed that up to 40% of the epileptic pediatric patients require polytherapy [3,4]. A German study demonstrated that polytherapy consisting of more than two AEDs was over two-fold more often in children and adolescents than in the patients over 65 years of age [3].

The problem of epileptic seizures affects everyday life of children and their social functioning. Seizures also significantly disrupt the course of the psychomotor development and the educational performance. Pharmacotherapy with AEDs has an impact on a number of biochemical processes in the body. Literature data indicate an association between epilepsy pharmacotherapy (i.e., the use of specific AEDs and the treatment duration) and the levels of biochemical parameters (including lipids, homocysteine (HCys) or folic acid) that may increase the risk of cardiovascular diseases. Data on the impact of AED treatment and lipid levels in the pediatric population are scarce and most often conflicting [5,6] and for the most part performed in patient groups whose epilepsy is controlled in monotherapy. Increased levels of HCys are observed in over 15% of children treated with AEDs, and the risk of hyperhomocysteinemia was suggested to increase during polytherapy [7]. 

Data on the association between the AEDs therapy and the activity of oxidative and antioxidant enzymes are not common and are mostly based on adult patients. The Turkish study performed in a group of 68 patients treated with AEDs showed that regardless of whether the treatment was mono or polytherapy, oxidative stress can be demonstrated in patients two months after its implementation. It has also been shown that the concentration of glutathione in patients receiving AEDs was significantly higher compared to the patients from the control group [8]. 

In addition, the long-term AEDs therapy may affect cognitive functions and behavior, thus knowledge on the role of individual AEDs in this matter may be useful in selection of the most suitable drugs for epileptic patients and by consequence in supporting their quality of life. However, data remain controversial. The most recent study by Operto et al. [9] performed in children receiving levetiracetam (LEV) for 2 years demonstrated mild improvement in overall cognitive abilities as well as in verbal skills, visual-perceptual reasoning, working memory, and processing speed. On contrary, carbamazepine (CBZ) may lead to deteriorations in cognitive functioning [10].

Lack of sufficient data from the pediatric population prompted us to assess and to compare levels of lipid parameters, as well as concentrations of selected aminothiols (i.e., HCys, cysteine, and glutathione) between epileptic children treated with multiple AEDs for at least 6 months and children without epilepsy. As to our knowledge, this pilot study is the first research analyzing levels of cysteine and glutathione in the pediatric population. 

## 2. Materials and Methods

### 2.1. Study Groups

In the present study, 44 children were prospectively recruited by the pediatric neurologist (IK) at the Department of Pediatric Neurology, Medical University of Silesia in Katowice (Poland), including 21 children with epilepsy treated with two or more AEDs and 23 children who received no pharmacological treatment with AEDs. Table 1 shows drug combination in of all patients receiving multiple treatment.

Inclusion criteria for the study group were as follows:

- Diagnosis of epilepsy based on the clinical picture and on the results of additional tests, especially electroencephalography and neuroimaging tests: magnetic resonance imaging (MRI) and/or computed tomography (CT) of the head;
- Patient’s age below 18 years;
- Polytherapy with AEDs for at least 6 months.

Exclusion criteria from the study group were as follows:

- Monotherapy;
- Non-epileptic seizures, no reliable diagnosis of epilepsy;
- Diagnosis of cardiovascular diseases (arterial ischemic stroke, hypertension, heart diseases).

The control group was recruited among children without seizures, hospitalized with mild to moderate head injuries.

Criteria for exclusion from the control group:

- History of seizure events (epileptic and non-epileptic);
- Treatment with AEDs for reasons other than epilepsy (i.e., behavioral problems, sleep disturbances, migraine).

The study protocol complied with the ethical guidelines of the 1975 Declaration of Helsinki and was approved by the Ethics Committee (no. of approval: PCN/0022/KB1/43/20). Written informed consent was obtained from parents of each recruited individual.

### 2.2. Biochemical Analyses

The concentrations of the parameters selected for analyses were assessed in the blood serum. Biological samples of antecubital venous blood were collected after 12 h of fasting, and then the samples were centrifuged within 2 h after being drawn. 

#### 2.2.1. Levels of Lipid Parameters 

Lipid parameters, i.e., total cholesterol (TC), high density lipoprotein (HDL) cholesterol (auto-HDL), low density lipoprotein (LDL) cholesterol (auto-LDL) and triglycerides (TG) were determined spectrophotometrically (VIS spectrophotometer DR 3900 HACH LANGE) with the use of commercially available kits (Pointe Scientific).

In addition, the levels of non-HDL cholesterol (subtracting HDL cholesterol from TC) and a very-low density lipoprotein (VLDL) level (20% of TG level) [11] were calculated. Additionally, the ratios of TC/HDL, TG/HDL and LDL/HDL were established. 

Based on the analysis of the lipid profile, each recruited case was assigned the status: dyslipidemia (1)/no dyslipidemia (0) and hypertriglyceridemia (1)/no hypertriglyceridemia (0). Both dyslipidemia and hypertriglyceridemia were established according to Sultan et al. [12] recommendations, i.e., dyslipidemia—either TC ≥ 200 mg/dL, or HDL < 40 mg/dL, or non-HDL ≥ 145 mg/dL and hypertriglyceridemia—for children aged up to 9 years TG ≥ 100 mg/dL, and for children aged 10–19 years, TG ≥ 130 mg/dL. As for lipid ratios we used the following intervals: TC/HDL normal <4, borderline 4–5, high >5; TG/HDL normal <3, and above normal >3; LDL/HDL normal <3, borderline 3–4, high >4.

#### 2.2.2. Analysis of Selected Aminothiols

The concentration of HCys was determined using the high-performance liquid chromatography (HPLC) method described earlier [13]: to 200 µL of the blood serum, 50 µL of the internal standard and 50 µL of tri-n-butylphosphine were added to cleave the disulfide bridges. After the derivatization of thiols with 7-fluorobenzene-2-oxa-1,3-diazole-4-sulfonate (SBD-F), the obtained combinations were separated on a LiChrospher 100 RP 18 chromatographic column, 250 × 4 mm ID, 5 µm (Merck). The separation was carried out in the reverse phase, in a concentration gradient. Phase A consisted of 0.1 µmol/µL acetate buffer solution (pH 4.0) containing 2% methanol (*v/v*); phase B consisted of 0.1 µmol/µL phosphate buffer solution (pH 6.0) containing 5% methanol (*v/v*). A linear gradient was applied from phase A to phase B, over 20 min (0–100%). Detection was performed at room temperature using a fluorescence detector (excitation wavelength 385 nm; emission wavelength 515 nm).

**Figure 1:**
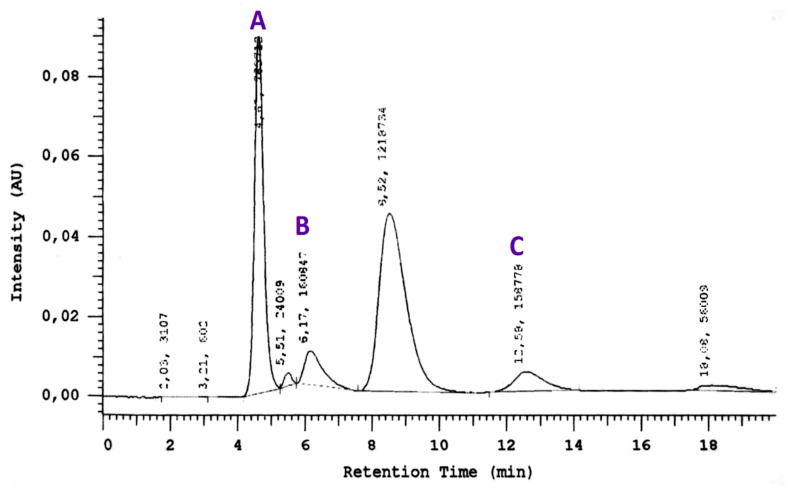
A representative chromatogram with the concentration–AUC plots for cysteine (**A**), homocysteine (**B**) and glutathione (**C**).

To demonstrate the linearity of the method, to draw calibration graphs and to determine the analytical measurement range serial dilutions for HCys, glutathione and cysteine were made. The area under the curve (AUC) of the peak retention times were calculated from the chromatograms and concentrations of HCys, cysteine, and glutathione from the corresponding concentration-AUC plots. A representative chromatogram and the concentration-AUC plots for HCys, cysteine, and glutathione is shown in Figure 1.

### 2.3. Statistical Analyses

For statistical analyses, STATISTICA 13.0 software (STATSOFT; Statistica, Tulsa, OK, USA) was used. The mean values (M) and the standard deviations (SD) were estimated for the continuous variables, while for the categorical variables the absolute numbers (*n*) and the relative numbers (%) were estimated. The normality of the data was verified with the Shapiro–Wilk test and on the basis of the visual assessment of the histograms. Due to the very small size of the groups, comparisons of the quantitative data between analyzed patients with epilepsy and patients without epilepsy were performed with the use of non-parametric *U* Mann–Whitney test throughout the study. Stochastic independence χ^2^ test with Yates’s correction was used to compare the categorical variables between total groups. In addition, Pearson’s correlation coefficients between lipid parameters and selected aminothiols were estimated. The result was considered to be statistically significant when the *p* value was below 0.05.

## 3. Results

### 3.1. General Characteristics of the Study Groups

The mean age of the analyzed cases as well as percentage of girls and boys did not differ between the two groups. Age of the patients at epilepsy onset was 2.46 years on average while treatment of epilepsy lasted 4.60 years on average. Over 71% of the epileptic patients had negative family history of epilepsy. Only three of the analyzed epileptic children were not burdened with other disorders (i.e., 14.28%). Some of the patients were diagnosed with the following comorbidities: autism, Asperger syndrome, tuberous sclerosis, Prader–Willi syndrome, Dravet syndrome, and ischemic-hypoxic encephalopathy (38.09%). One of the patients presented the condition after a history of Lyell’s syndrome, and the other one after limbic encephalitis. Almost 86% of the patients presented mild or severe mental retardation. The general characteristics of the epileptic patients and controls are shown in Table 2. 

### 3.2. Levels of Lipid Parameters in Analyzed Groups

The mean concentrations of lipid parameters in epileptic children and controls are shown in Table 3. No differences in mean levels of lipids as well as in mean levels of lipid ratios were observed between the groups. No differences in lipid concentrations were also observed for girls and boys subgroups. 

Percentage of dyslipidemic and hypertriglycerydemic cases also did not differ between the two groups. Similarly, the frequencies of particular intervals for lipid ratios were comparable between the patients and controls. 

### 3.3. Levels of Aminothiols in Analyzed Groups

In the whole analyzed groups, neither the level of HCys nor the level of cysteine differed between the patients and the control group. However, we demonstrated a significantly lower level of glutathione in children with epilepsy compared to children without epilepsy (*p* < 0.001). Table 4 demonstrates mean levels of the selected aminothiols in total groups as well as in sex subgroups. 

Epileptic boys presented slightly higher levels of all analyzed aminothiols than epileptic girls but the differences were not significant. Similarly, differences in the levels of HCys, cysteine and glutathione were not significant between boys and girls without epilepsy. On the other hand, glutathione level was lower in boys with epilepsy than in boys without epilepsy (*p* = 0.007). In addition, epileptic boys showed also a more elevated level of cysteine than boys from the control group, but the difference was close to the bound of significance (*p* = 0.098). HCys concentration was slightly higher in epileptic boys than in boys without epilepsy but without significance. The level of glutathione was also statistically decreased in epileptic girls compared to girls without epilepsy (*p* = 0.006). The mean levels of HCys and cysteine did not differ between girls with and without epilepsy.

### 3.4. Correlations between Levels of Lipids and Aminothiols in Analyzed Groups

Correlation coefficients between lipids and analyzed aminothiols in epileptic children and controls are presented in Table 5. In children with epilepsy, TG and VLDL concentrations correlated positively with the level of cysteine. The higher the levels of TG and VLDL, the greater the level of cysteine on average.

For epileptic girls, no significant correlations were observed between lipids and aminothiols. In epileptic boys, the positive correlation between the level of TC and the level of HCys was observed (*r* = 0.549, *p* = 0.052). In turn, in boys from the control group the level of HCys was positively correlated with the following lipid parameters: TG and VLDL (*r* = 0.562, *p* = 0.029 for each) and non-HDL (*r* = 0.602, *p* = 0.018). On the other hand, in girls without epilepsy, a strong negative correlation (*r* = −0.835, *p* = 0.020) between the level of non-HDL and the level of cysteine was found.

## 4. Discussion

In the present study, we observed no association between long-term therapy using multiple AEDs and lipid levels. Our results are consistent with the previous study by Deda et al. [14]. However, in the Turkish study, the patients were younger than ours and they were treated in monotherapy with CBZ. In addition, the authors studied a sample with a smaller number of cases [14]. In turn, the study based on Greek children with epilepsy demonstrated significant reduction in the levels of TG and lipid ratios, i.e., TG/HDL and LDL/HDL during the 12 months of epilepsy therapy using LEV [5]. On the other hand, children from India treated with phenytoin (PHT) for at least 6 months had significantly higher levels of TC, HDL, LDL and TG compared to healthy children [6]. In children treated with oxcarbazepine (OXC), increased the levels of TC, HDL and TG were found, while OXC did not increase the level of LDL. It was also suggested that the treatment with VPA and LEV did not influence the lipid levels in epileptic children compared to the healthy group [6]. 

In our study, the mean levels of HCys were comparable between epileptic children and controls. We assumed that values for optimal HCys plasma level were as follows: for children under 15 years of age, 10 µmol/L, and for children over 15 years of age, 15 µmol/L [15]. Using these cut-off points, we observed no difference in the frequency of hyperhomocysteinemic cases between epileptic children and children without epilepsy. 

Previous studies demonstrated that the level of HCys in patients treated for epilepsy may increase [7], and this elevation is likely due to the altered remethylation process of HCys to methionine [16]. Long-term therapy of AEDs and the anticonvulsant drug itself may influence the levels of HCys to different extents. The French study demonstrated that patients receiving CBZ for at least one month had significantly higher concentrations of plasma HCys than those receiving OXC [16]. The results of the meta-analysis confirmed that CBZ and VPA treatment may cause an elevation in the serum levels of HCys [17]. In the human body, HCys is synthesized in all types of cells and it participates mainly in oxidation–reduction reactions and thiol–disulfide exchange. HCys contains a highly reactive thiol group and can modify the activity of the proteins by forming disulfide bonds in reaction either with cysteine side chain residues or with amino groups [18]. HCys cause severe oxidative stress that stimulates the production of pro-inflammatory cytokines and may also result in chronic inflammation. Elevated level of HCys is an established risk factor for coronary heart disease in adults as well as for vascular disease in developmental age. Moderate hyperhomocysteinemia is associated with a 4-fold increase in the risk of ischemic diseases in children [19]. In adults, elevated HCys levels in the acute phase of ischemic stroke was suggested to be a risk factor for mortality [20].

Data on the effect of AEDs on the level of HCys coming from pediatric studies show contradictory findings [21,22]. Kumar et al. [21] did not observe elevation in the mean level of HCys during six months of the treatment with CBZ (HCys level at recruitment: 11.51 ± 3.95 µmol/L vs. HCys level at 6 months: 11.77 ± 6.65 µmol/L). However, the authors demonstrated that the frequency of children with the level of HCys above 15 µmol/L increased from 16% to 27% after 6 months of the monotherapy with CBZ [21]. On the other hand, the mean level of HCys was higher in Italian children with epilepsy treated in mono or polytherapy than the mean concentrations of HCys in the control group [22]. In our epileptic boys, the level of HCys positively correlated with the level of TC (*r* = 0.549), although the result was on bound of significance. The higher the level of HCys, the greater the level of TC on average. The study by Coppola et al. [22] reported that the elevation in the level of HCys is related to low levels of folate.

In the present study, glutathione level was the only parameter that significantly differed between the two groups. The mean level of glutathione was lower in epileptic children receiving multi-drug therapy than in children without epilepsy. We also observed that higher levels of glutathione differentiated boys with epilepsy from boys without epilepsy as well as epileptic girls from control girls. In contrary to the present results, glutathione levels were observed to be higher, although not significantly, in adult patients with epilepsy than in controls [8]. Similarly, in a Turkish study based on a cohort with idiopathic epilepsy also including adolescents, glutathione was significantly increased in the epileptic cases compared to the controls [23]. 

Glutathione is an important metabolic antioxidant compound which prevents the formation of free radicals in many tissues and protects cells from oxidative damage by reducing the disulfide groups of cellular molecules or by scavenging free radicals [24]. Previously, it was demonstrated that increased generation of free radicals or reduced activity of antioxidative defense mechanisms can result in appearance of some types of seizures and thus may increase the risk of the seizure recurrence [25]. Another study from Turkey by Yüksel et al. [25] based on pediatric patients with epilepsy showed that the antioxidant systems are better regulated in patients on CBZ therapy than in epileptic children treated with VPA. The authors observed opposite results on glutathione peroxidase, superoxide dismutase and the serum lipid peroxidation when CBZ or VPA was used. Combinations of AEDs tend to deplete hepatic stores of glutathione which results in vulnerability of hepatocytes to injury from free radicals [26]. In adult patients, the level of glutathione was significantly decreased in Chinese women with epilepsy treated in monotherapy with PHT compared to controls [27]. 

In our study, cysteine concentrations were comparable between the two analyzed groups. However, we found higher, but insignificant levels of cysteine in epileptic boys than in boys from the control group. No such relation was observed in the girl subgroups. In turn, concentrations of TG and VLDL correlated positively with the level of cysteine. The higher the levels of TG and VLDL the greater the level of cysteine on average. In a study by Ramazan et al. [23], cysteine was significantly higher in controls than in epileptic patients and especially low levels of cysteine was observed in a group of patients taking LTG. 

We are aware of several limitations of the present study. First, the number of analyzed cases is low. Second, the analyzed epilepsy group is very heterogenous as children were treated with different AEDs which may show different effects on the levels of lipids and HCys. Therefore, we were not able to make a division into subgroups according to specific combinations of drugs. However, this research project was performed during the pandemic situation which resulted in limited number of scheduled hospital admissions. Third, the group without epilepsy was recruited among children hospitalized with mild to moderate head injuries, and previous studies based on adults showed that head injury may affect the parameters evaluated in our study [28]. Previously, it was shown that traumatic brain injury alters DNA and histone methylation and causes oxidative stress. Under such conditions, HCys enters the transsulfuration pathway to generate glutathione, which is an important antioxidant [29]. The weaknesses of our study also include the wide range of treatment duration, as well as the diversity of epilepsy etiology, which may affect the metabolism of anticonvulsants. However, our study is the first research on the relationship between multi-drug therapy with AEDs and the levels of selected aminothiols (HCys, glutathione and cysteine) in pediatric patients with epilepsy. 

## 5. Conclusions

The most important result obtained in our study is lower mean concentration of glutathione in epileptic children compared to children without epilepsy. This indicates the oxidative stress of patients receiving multiple AEDs, which in turn may affect their well-being, the function of the liver, and the condition of the cardiovascular system due to long-term treatment. Therefore, it would be worth planning not only to study groups of children with epilepsy homogenous for age and treated with the same drugs, but also to conduct long-term observation of indicators showing the state of oxidative stress in these children. 

The results obtained in this way could form the basis of preventive actions aimed at protection against the distant effects of oxidative stress experienced in childhood due to antiepileptic therapy that could be experienced in adulthood. On the other hand, it can be theoretically assumed that the state of oxidative stress in a given patient will increase, but also compensatory mechanisms will be activated and the observation carried out for many months may show the return of biochemical features indicating stress to the normative values.

## Figures and Tables

**Table 1:** Combination of AEDs and morphology of seizures at both onset and follow-up in analyzed epileptic children.

Patient	Combination of Drugs	Morphology of Seizures at Both Onset and the Follow-Up
1	LEV, LTG	generalized seizures; focal aware seizures; consciousness disturbances
2	VPA, LEV, CLB	generalized tonic-clonic seizures; atonic seizures
3	VPA, LEV, CZP	generalized tonic-clonic seizures; tonic seizures; consciousness disturbances
4	VPA, VGB	polymorphic seizures; myoclonic; consciousness disturbances
5	TPM, LEV	generalized seizures
6	LEV, PHB	generalized seizures
7	VPA, TPM, VGB	polymorphic seizures
8	VPA, CLB	generalized seizures
9	LEV, LTG	polymorphic seizures; myoclonic; tonic-clonic seizures
10	VPA, CLB	generalized tonic-clonic seizures
11	VPA, CLB, STP, LEV	generalized seizures
12	CLB, LTG, LEV	generalized clonic seizures
13	PHB, LTG, LEV	polymorphic seizures; simple partial seizures
14	VPA, LEV	myoclonic
15	LEV, CZP, LTG	generalized seizures; consciousness disturbances
16	VPA, LEV	generalized seizures
17	VPA, LEV	generalized seizures; tonic seizures
18	CZP, LTG, TPG	tonic-clonic seizures; myoclonic
19	VPA, LEV	polymorphic seizures; myoclonic
20	VPA, LEV	generalized seizures
21	VPA, LEV	generalized seizures

PHT—phenytoin; PHB—phenobarbital; VPA—valproate; VGB—vigabatrin; LEV—levetiracetam; TPM—topiramate; LTG—lamotrigine; CZP—clonazepam; CLB—clobazam; STP—stiripentol.

**Table 2:** Demographic and clinical characteristics of the analyzed groups of patients.

Variable	
Age (years), M ± SD Epileptic patients Controls	7.13 ± 4.40 7.59 ± 3.84
Sex (F/M) Epileptic patients Controls	8/13 7/16
Age at epilepsy onset (years), M ± SD Epileptic patients	2.46 ± 2.40
Duration of treatment (years), M ± SD Epileptic patients	4.60 ± 4.28
Positive family history of epilepsy, n (%) Epileptic patients	6 (28.57)
The presence of comorbidities, n (%) Epileptic patients	18 (85.72)

M—mean; SD—standard deviation; F—female; M—male.

**Table 3:** Levels of lipid parameters in analyzed groups.

Lipid Parameters	Epileptic Children *N* = 21	Controls *N* = 23	*p*
TC (mg/dL), M ± SD	128.75 ± 26.08	129.31 ± 26.08	0.852
LDL (mg/dL), M ± SD	102.24 ± 28.20	103.79 ± 40.04	0.658
HDL (mg/dL), M ± SD	63.21 ± 15.41	62.22 ± 12.43	0.907
TG (mg/dL), M ± SD	103.70 ± 47.29	88.47 ± 42.48	0.243
VLDL (mg/dL), M ± SD	20.74 ± 9.46	17.69 ± 8.50	0.243
Non-HDL (mg/dL), M ± SD	65.54 ± 29.83	67.09 ± 24.37	0.889
TC/HDL, M ± SD	2.15 ± 0.66	2.14 ± 0.57	0.852
LDL/HDL, M ± SD	1.73 ± 0.73	1.71 ± 0.67	0.963
TG/HDL, M ± SD	1.71 ± 0.87	1.49 ± 0.79	0.339

M—mean; SD—standard deviation; TC—total cholesterol; LDL—low density lipoprotein; HDL—high density lipoprotein; VLDL—very low density lipoprotein; TG—triglycerides. *p* for U Mann–Whitney test.

**Table 4:** Levels of selected aminothiols (HCys, cysteine and glutathione) in total groups and sex subgroups.

Lipid Parameters	Epileptic Children	Controls	*p*
Total groups, *N*	21	23	
HCys (µmol/L), M ± SD	9.13 ± 3.34	8.47 ± 2.49	0.430
Cysteine (µmol/L), M ± SD	194.64 ± 33.96	189.47 ± 35.08	0.585
Glutathione (µmol/L), M ± SD	1.49 ± 0.35	2.39 ± 1.17	**<0.001**
Female subgroups, *N*	8	7	
HCys (µmol/L), M ± SD	7.80 ± 3.42	8.01 ± 2.47	0.694
Cysteine (µmol/L), M ± SD	176.58 ± 34.48	193.31 ± 43.86	0.370
Glutathione (µmol/L), M ± SD	1.41 ± 0.12	2.74 ± 1.88	**0.006**
Male subgroups, *N*	13	15	
HCys (µmol/L), M ± SD	9.96 ± 3.14	8.69 ± 2.56	0.185
Cysteine (µmol/L), M ± SD	205.76 ± 29.61	187.68 ± 31.79	0.098
Glutathione (µmol/L), M ± SD	1.54 ± 0.43	2.22 ± 0.65	**0.007**

M—mean; SD—standard deviation; HCys—homocysteine; *p* for U Mann–Whitney test. Significant difference is in bold.

**Table 5:** Correlation coefficients between the levels of lipids and aminothiols (HCys, cysteine and glutathione) in analyzed groups.

Lipid Levels	Epileptic Children											
	TC (mg/dL)		TG (mg/dL)		HDL (mg/dL)		LDL (mg/dL)		Non-HDL (mg/dL)		VLDL (mg/dL)	
AMINOTHIOLS	r	*p*	r	*p*	r	*p*	r	*p*	r	*p*	r	*p*
HCys (µmol/L)	0.134	0.545	0.215	0.349	0.014	0.953	0.162	0.483	0.118	0.610	0.215	0.349
Cysteine (µmol/L)	−0.370	0.099	0.461	0.035	−0.014	0.953	0.176	0.445	−0.324	0.152	0.461	0.035
Glutathione (µmol/L)	0.029	0.900	−0.259	0.256	−0.102	0.659	−0.398	0.074	0.079	0.734	−0.259	0.256
**Lipid Levels**	**Controls**											
	**TC** **(mg/dL)**		**TG** **(mg/dL)**		**HDL** **(mg/dL)**		**LDL** **(mg/dL)**		**Non-HDL** **(mg/dL)**		**VLDL** **(mg/dL)**	
AMINOTHIOLS	r	*p*	r	*p*	r	*p*	r	*p*	r	*p*	r	*p*
HCys (µmol/L)	0.275	0.216	0.287	0.194	0.011	0.961	0.186	0.406	0.290	0.190	0.287	0.194
Cysteine (µmol/L)	−0.131	0.562	0.317	0.150	−0.214	0.339	−0.330	0.134	−0.025	0.913	0.317	0.150
Glutathione (µmol/L)	−0.129	0.567	−0.140	0.534	−0.174	0.439	−0.056	0.806	−0.045	0.842	−0.140	0.534

M—mean; SD—standard deviation; HCys—homocysteine; TC—total cholesterol; LDL—low density lipoprotein; HDL—high density lipoprotein; TG—triglycerides; VLDL—very low density lipoprotein. r—Pearson’s correlation coefficient. Significant differences are in bold.

## Data Availability

The data presented in this study are available on request in the Department of Basic Biomedical Science, Faculty of Pharmaceutical Sciences, Medical University of Silesia in Katowice (Poland). The data are not publicly available due to privacy restrictions.
